# A Highly Sensitive Electrochemical Sensor for Capsaicinoids and Its Application in the Identification of Illegal Cooking Oil

**DOI:** 10.3390/bios13090863

**Published:** 2023-09-01

**Authors:** Wenjing Lyu, Min Ding, Ying Zhou, Mengdan Jiang, Yanru Li, Yanxiang Ding, Zhong Zhang, Xue Wei, Xiaoqing Zhang

**Affiliations:** 1 Key Laboratory of Clinical Laboratory Diagnostics, Ministry of Education of China, School of Laboratory Medicine, Chongqing Medical University, Chongqing 400016, China; 305452@hospital.cqmu.edu.cn (W.L.); dingmin@cqmu.edu.cn (M.D.); 17774964041@163.com (Y.Z.); 18208371513@163.com (M.J.); 18468154207@163.com (Y.L.); 2022110551@stu.cqmu.edu.cn (X.W.); 2 The First Clinical College, Chongqing Medical University, Chongqing 400016, China; tanktank0905@163.com; 3 Material Evidence Identification Center, Chongqing Public Security Bureau, Chongqing 400016, China; zhangzhong5123@126.com

**Keywords:** capsaicinoids, illegal cooking oil, screen-printed carbon electrode, composite nanoparticles, diazotization–coupling reaction

## Abstract

Capsaicinoids, mostly from chili peppers, are widely used in daily life. Capsaicinoids are considered to be markers for the identification of illegal cooking oil (ICO), which is a serious threat to public health. The identification of capsaicinoids can help reveal food-related fraud, thereby safeguarding consumers’ health. Here, a novel and ultrasensitive method was established with a signal amplification strategy for the detection of capsaicinoids. AuNPs@Fe_3_O_4_ nanocomposites were functionalized with 4-aminothiophenol (4-atp). After diazotization, 4-atp on AuNPs@Fe_3_O_4_ reacted with capsaicinoids and formed capsaicinoids-azo-atp-AuNPs@Fe_3_O_4_. Ultimately, capsaicinoids-azo-atp-AuNPs@Fe_3_O_4_ was dropped onto the surface of a screen-printed carbon electrode (SPCE) and detected via the differential pulse voltammetry (DPV) method. AuNPs@Fe_3_O_4_ nanocomposites increased the specific surface area of the electrode. Moreover, the diazotization–coupling reaction enriched the analytes on the electrode surface. Liquid–liquid extraction was used for sample pretreatment. Under a pH value of 9.0 and concentration of 0.20 mol/L for the supporting electrolyte, the linearity of capsaicinoids in ICO is from 0.10 to 10.00 ng/mL, and the limit of detection (S/N = 3) is 0.05 ng/mL. This method is ultra-sensitive, reliable, and cost-effective for the detection of capsaicinoids. Herein, this method provides a promising tool for the identification of ICO.

## 1. Introduction

In recent years, cooking oil safety accidents have occurred frequently in developing countries, increasing the attention devoted toward food safety inspections [1]_._ Illegal cooking oil (ICO) is a general term for various inferior oils. Waste cooking oil (WCO) from the catering industry is the main source of ICO [2]. It is estimated that more than five million tons of WCO are produced each year in the catering industry in large and medium cities in China [3,4]. WCO is added into edible vegetable oil by some mercenary merchants for the purpose of being sold, which is a serious threat to public health. After the refining of ICO, the essential fatty acids and plant sterols are greatly reduced, accompanied by the production of a large amount of hazardous substances such as mycotoxin. After refining and purification, the quality of ICO is often close to the national hygienic standard of edible vegetable oil in terms of sensory index and conventional properties. It is difficult for consumers and government supervisors to identify ICO solely via the senses of sight and smell [5,6]. This phenomenon is particularly serious in China. Therefore, distinguishing ICOs from edible oils in order to cope with the subtle adulterations appears to be especially important.

As large quantities and diverse types are consumed, chili pepper fruits are commercially important. Most chili peppers taste pungent due to a group of lipophilic alkaloids called capsaicinoids [7,8,9]. The most abundant ingredients of these compounds are capsaicin (trans-8-methyl-N-vanillyl-6-nonenamide) and dihydrocapsaicin (8-methyl-N-vanillyl-nonanamide), which are responsible for about 90% of the fruits’ spiciness [10,11]. Capsaicinoids are flavoring agents that are added to some extent to three meals a day, and they are active ingredients that are inevitably present in ICO due to their own lipophilicity [2]. Therefore, capsaicinoids are considered to be markers for the identification of ICO. A simple, rapid, and sensitive method is urgently required for the determination of capsaicinoids, providing technical support for the identification of ICO. 

In recent years, some liquid chromatography-mass spectrometry (LC-MS/MS) methods have been reported for the determination of capsaicinoids in ICO. Wang’s group [12] explored an LC-MS/MS method to detect trace residues of three capsaicinoids (capsaicin, dihydrocapsaicin, and nonylic acid vanillylamide) in ICO. The ICO samples were pretreated via liquid–liquid extraction (LLE) combined with solid–phase extraction. Zhao’s group [13] developed an ultra-high-performance liquid chromatography (UPLC)—MS/MS method for capsaicin and dihydrocapsaicin, with solid phase extraction for sample pretreatment. These methods achieved the separation of capsaicinoids. However, they required complicated procedures, long sample pretreatment time, and expensive instruments, which made them unsuitable for the analysis of capsaicinoids in ICO in the food hygiene field. 

Recently, electrochemical sensors have attracted significant interest in research because of their advantages, such as high sensitivity, rapid detection, and potential miniaturization [14,15,16]. Electrochemical detection methods were also used for the determination of capsaicinoid concentration in related research [17,18,19,20,21]. The screen–printed carbon electrode (SPCE) has the characteristics of a simple procedure, convenient carrying, a low price, and the ability to be mass produced [18,21,22]. The surface modification of the electrode with nanomaterials increases the specific surface area of the electrode significantly [23]. Moreover, nanomaterials have the properties of excellent adsorption and catalytic activity, which can improve the sensitivity of detection remarkably. Gold nanoparticles (AuNPs) possess excellent conductivity, a large surface area, good stability, and biocompatibility. As a result, they have been shown to have extensive applications in the field of nanosensors, enabling high sensitivity and the rapid detection of target substances [24]. They have significant potential application in areas such as biomedicine, environmental monitoring, and food safety [25,26,27]. Moreover, the surface of gold nanoparticles can be chemically modified to enable specific binding with target molecules. Due to their unique magnetic properties, Fe_3_O_4_ nanoparticles can be manipulated through an external magnetic field, which plays an important role in the pretreatment and enrichment of samples [28]. Specifically, during the sample preprocessing stage, Fe_3_O_4_ magnetic nanoparticles can rapidly separate the connected target molecules under the influence of an applied magnetic field, thereby enhancing the efficiency of sample extraction. For sample detection, Fe_3_O_4_ magnetic nanoparticles can fix the analytes connected on them to the electrode surface under the influence of an external magnetic field, markedly improving sensitivity [29]. Additionally, magnetic nanoparticles possess a substantial surface area and exceptional conductivity, which lead to a significant enhancement of the detection sensitivity for target analytes in various applications [30]. Gold magnetic particles (GMP), AuNPs immobilized onto the surface of magnetic nanoparticles to form AuNPs@Fe_3_O_4_ nanocomposites, provide a large specific surface area, good stability, outstanding magnetic separation performance, and good biocompatibility. Yang and co-workers [31] reported an electrochemical immunosensor for the rapid determination of clenbuterol using an SPCE modified via GS-Nf film and GMP-coated bovine serum albumin–clenbuterol conjugates based on a competitive immunoassay mode, with a detection limit of 0.22 ng/mL. Chen and co-workers [32] developed a highly sensitive method to detect the concentration of nitrite ions using GMPs via surface-enhanced Raman scattering.

Herein, an ultra-sensitive electrochemical method was established using AuNPs@Fe_3_O_4_ nanocomposites combined with the diazotization–coupling reaction for the detection of capsaicinoids in ICO via the SPCE. The study employed high-energy electron beam radiation to synthesize AuNPs@Fe_3_O_4_ nanocomposites, which were subsequently functionalized using Au-S bonds with 4-aminothiophenol (4-atp). Subsequently, capsaicinoids were immobilized on the 4-atp-modified Au/Fe_3_O_4_ surface through the diazonium–coupling reaction. Due to the significant surface area and good biocompatibility of AuNPs, the active sites of capsaicinoids were greatly increased when coated onto the composite beads’ surfaces. The application of an external magnetic field facilitated convenient, rapid, and non-chemically polluting separation of the product. Convenient, rapid, and nonchemical contamination separation of the product can be achieved via the use of an external magnetic field. LLE was carried out for the pretreatment of ICO samples. This study provides an ultra-sensitive and effective method for ICO identification in the food hygiene field.

## 2. Experimental Section

### 2.1. Reagents and Apparatus

Capsaicin (Cas No. 404-86-4, purity: ≥98%) and dihydrocapsaicin (Cas No. 19408-84-5, purity: ≥98%) were purchased from Shanghai Standard Biotechnology Co., Ltd. (Shanghai, China). Fe_3_O_4_ nanoparticles with a diameter of 20 to 30 nm were purchased from micromod Partikeltechnologie GmbH (Rostock, Germany). Gold(III) chloride trihydrate (HAuCl_4_·3H_2_O) was purchased from Sigma-Aldrich (St. Louis, MO, USA). 4-atp was purchased from Tokyo Chemical Industries Co., Ltd. (Tokyo, Japan). Polyvinyl alcohol (PVA) was purchased from Shanghai Sangon Biological Engineering Technology and Service Co., Ltd. (Shanghai, China). Other chemicals were of an analytical grade and were bought from Chongqing Company of Chemical Reagent (Chongqing, China). Ultrapure water was acquired from a Milli-Q system (Milford, MA, USA). Edible rapeseed oil was purchased from a supermarket, and was used as a blank oil sample to establish and evaluate the experimental method.

Electrochemical measurements were performed using a CHI852C electrochemical workstation from Shanghai Chenhua Instrument Co., Ltd. (Shanghai, China). Ultrasonic extraction was carried out using a KQ-3200DB from Kunshan Ultrasonic Instrument Co., Ltd. (Jiangsu, China). High-energy electron beam irradiation was carried out on a HEXTRON-3000 electronic accelerator from Nuctech Co., Ltd. (Beijing, China). UV–Vis spectroscopy experiments were performed on a Nano–Drop1000 from Thermo Fisher Scientific (Waltham, MA, USA). The morphology of the AuNPs@Fe_3_O_4_ nanocomposite was characterized via a Tecnai G2 F20 transmission electron microscope (TEM) from FEI (Hillsboro, OR, USA). A QTRAP 5500 mass spectrometer (AB Sciex, Concord, ON, Canada) was used for the LC–MS/MS analysis. The three-electrode SPCE was fabricated as reported [33], and consisted of a graphite working electrode with a diameter of 2.5 mm, a graphite auxiliary electrode, and an Ag/AgCl reference electrode.

### 2.2. Preparation of the AuNPs@Fe_3_O_4_ Composite Nanoparticles

AuNPs@Fe_3_O_4_ composite nanoparticles were synthesized via high-energy electron beam irradiation as reported, with a slight modification [34]. Fe_3_O_4_ nanoparticles were dispersed in an aqueous solution to prepare a 1.0 mg/mL Fe_3_O_4_ suspension containing 0.050 mmol/L HAuCl_4_, 10.0 mg/mL PVA, and 125.0 mmol/L 2-propanol. The suspension was sonicated for 15 min in an ultrasonic bath to achieve well-distributed Fe_3_O_4_ nanoparticles before the irradiation. This suspension was then irradiated at a dose of 6.0 kGy with a 10.0 MeV high-energy electron beam in a glass vial. The obtained AuNPs@Fe_3_O_4_ nanocomposites were collected with a magnet, washed with ultrapure water twice, and then resuspended in ultrapure water.

### 2.3. Sample Preparation

Six ICO samples were obtained from the Material Evidence Identification Center of Chongqing Public Security Bureau (Chongqing, China) and stored at −20 °C until analysis. The LLE was adopted for the sample preparation. Firstly, 150 μL of an oil sample and 150 μL of dichloromethane were added into a 2 mL tube and vortexed. Afterwards, 1.2 mL of 0.50 mol/L NaOH was added into the tube. Finally, the mixture was shaken severely at 250 rpm for 20 min and centrifuged at 4000 rpm for 10 min at 4 °C to obtain 900 μL of the upper layer solution. 

### 2.4. Capsaicinoids Reacted with 4-atp on the Surface of AuNPs@Fe_3_O_4_ Nanocomposites via the Diazotization–Coupling Reaction

**Scheme 1:**
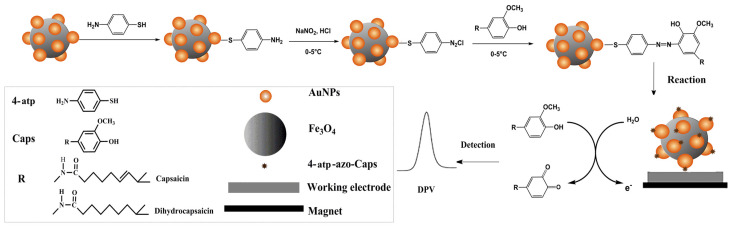
Schematic diagram for the diazotization-coupling reaction and electrochemical detection.

Capsaicinoids reacted with 4-atp on the surface of AuNPs@Fe_3_O_4_ nanocomposites via the diazotization–coupling reaction, as shown in Scheme 1. AuNPs@Fe_3_O_4_ nanocomposites were directly immersed into the ethanol solution containing 1.0 mmol/L 4-atp for 12 h to form atp-AuNPs@Fe_3_O_4_. The uncombined 4-atp was rinsed completely with ethanol and ultrapure water. Additionally, the suspension of atp-AuNPs@Fe_3_O_4_ was then stored at 4 °C in an amber laboratory bottle. Before the diazotization reaction, the atp-AuNPs@Fe_3_O_4_ complex was sonicated for 15 min. Subsequently, 1 mL of the atp-AuNPs@Fe_3_O_4_ suspension was taken out and washed with a 1.0 mmol/L HCl solution, and then removed to an ice-water bath, and finally, 100 μL of 10.0 mmol/L NaNO_2_ was slowly added in with a little excess to ensure the completeness of the diazotization reaction. The mixed solution was placed in a microplate fast shaker and incubated for 30 min at 2–4 °C. For the coupling reaction of capsaicinoids, 15 μL of the atp-AuNPs@Fe_3_O_4_ suspension after diazotization was added into the prepared sample solution, and concentrated hydrochloric acid solution was used to adjust the pH value of the mixture to 9.0. The coupling reaction lasted for 30 min at 2–4 °C with slight shaking. Finally, the capsaicinoids-azo-atp-AuNPs@Fe_3_O_4_ was rinsed with ultrapure water and then modified onto the surface of the SPCE. The modified electrode was dried at room temperature (23 ± 2 °C).

### 2.5. Electrochemical Measurement Procedures

The electrochemical measurement was performed using the DPV technique on a CHI852C electrochemical workstation connected to the SPCE. The potassium phosphate solution, 50 μL with a pH value of 9.0 as the supporting electrolyte, was added to the modified electrode. The experimental detection voltage was set from 0.2 V to 0.9 V, with a pulse period of 0.2 s, a pulse amplitude of 100 mV, and a pulse width of 0.05 s. All measurements were conducted at a temperature of 23 ± 2 °C.

### 2.6. The Principle behind the Electrochemical Detection for Capsaicinoids

The principle behind the electrochemical detection for capsaicinoids was elucidated in our previous work [22]. In summary, cyclic voltammetry (CV) is used to analyze the electrochemical behavior of capsaicin compounds. During the first scan, an irreversible step occurs involving the oxidation of phenolic hydroxyl groups and the hydrolysis of methoxy groups, as well as the formation of an o-benzoquinone unit. Subsequently, during the second scan, ortho-benzoquinone participates in the oxidation–reduction electrochemical cycle along with an o-hydroxyphenol group. Only the irreversible oxidation appeared when detected via the differential pulse voltammetry (DPV) method.

## 3. Results and Discussion

### 3.1. The Characterization of AuNPs@Fe_3_O_4_ Nanoparticles

**Figure 1:**
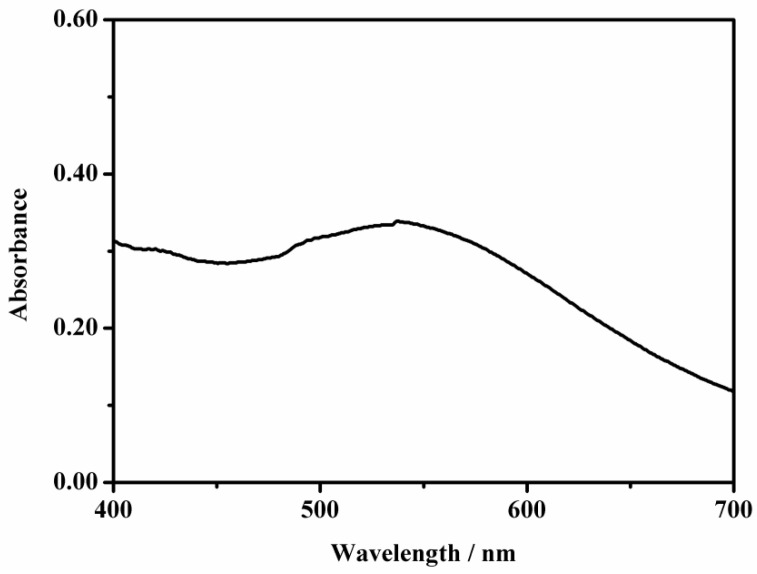
The UV-Vis characterization of the AuNPs/Fe_3_O_4_ nanocomposites.

The principle and procedures for the synthesis of AuNPs@Fe_3_O_4_ composite nanoparticles were reported by Satoshi and co-workers [34]. The synthesized AuNPs@Fe_3_O_4_ nanocomposites were characterized via ultraviolet and visible spectroscopy, as shown in Figure 1. A wide peak appeared at around 545 nm in the absorption spectrum, indicating that AuNPs were successfully embedded in Fe_3_O_4_ particles. 

**Figure 2:**
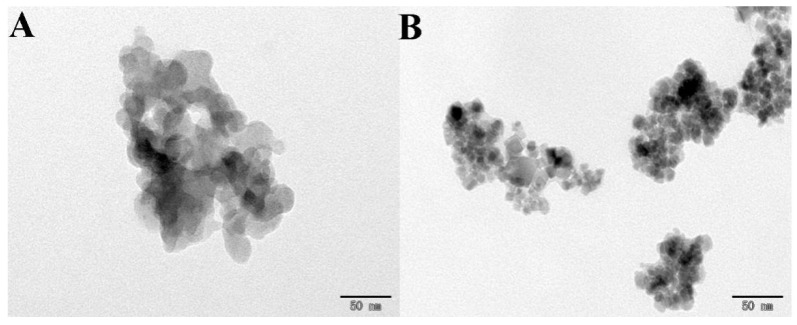
The TEM image of the surface morphology: (**A**) Fe_3_O_4_ nanoparticles. (**B**) AuNPs/Fe_3_O_4_ nanocomposites.

In addition, the morphology of the AuNPs@Fe_3_O_4_ nanocomposites was characterized using a transmission electron microscope (TEM), as shown in Figure 2. An image of the magnetic Fe_3_O_4_ particles is shown in Figure 2A, and an image of the nanocomposite after coating the AuNPs is shown in Figure 2B. The results revealed that AuNPs were well coated on the surface of the magnetic Fe_3_O_4_ particles. This composite provides a large specific surface area and enhances the stability as well as dispersibility of the magnetic Fe_3_O_4_ particles.

### 3.2. Electrochemical Behavior of Capsaicinoids

**Figure 3:**
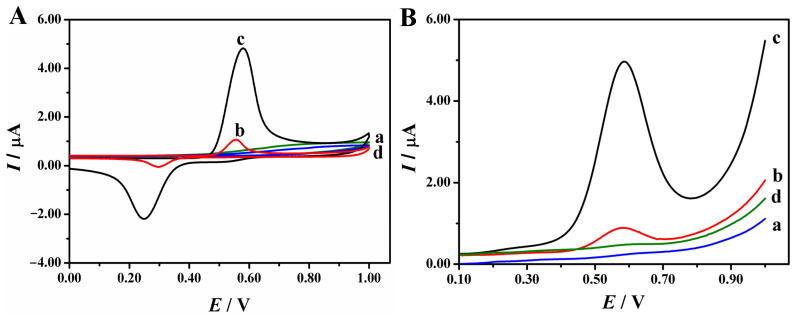
Electrochemical behavior of capsaicinoids on the SPCE. (**A**) The CV curves in the supporting electrolyte for 3.0 ng/mL capsaicin on (a) the bare SPCE; (b) AuNPs/Fe_3_O_4_-modified SPCE; (c) capsaicinoids-azo-atp-AuNPs/Fe_3_O_4_-modified SPCE; and for blank solution on (d) the capsaicinoids-azo-atp-AuNPs/Fe_3_O_4_-modified SPCE. (**B**) The DPV curves in the supporting electrolyte for 3.0 ng/mL capsaicin on (a) the bare SPCE; (b) AuNPs/Fe_3_O_4_-modified SPCE; (c) capsaicinoids-azo-atp-AuNPs/Fe_3_O_4_-modified SPCE; and for blank solution on (d) the capsaicinoids-azo-atp-AuNPs/Fe_3_O_4_-modified SPCE.

The electrochemical behavior of capsaicin was characterized via CV at a scan rate of 0.1 V/s, as shown in Figure 3A. No obvious peaks were observed when 3.0 ng/mL capsaicin was added onto the bare SPCE (curve a). The prepared AuNPs@Fe_3_O_4_ nanocomposites were sonicated for 15 min, and then 5 μL of the dispersion was dropped onto the surface of the SPCE. Next, the modified electrode was dried at room temperature. A weak oxidation current was observed when a 3.0 ng/mL capsaicin solution was added to the above modified SPCE (curve b). This indicated that the conductivity was enhanced because of the increased specific surface area of the electrode after modification with AuNPs@Fe_3_O_4_ nanocomposites. Finally, capsaicin was combined with AuNPs@Fe_3_O_4_ nanocomposites via the diazotization–coupling reaction on a self-assembled 4-atp monolayer, and it was added to the SPCE for detection. The results showed that the oxidation peak current was significantly increased (curve c). In addition, the blank solution was detected based on AuNPs@Fe_3_O_4_ nanocomposites combined with the diazotization–coupling reaction. Inapparent oxidation peaks were discovered (curve d). The results indicated that the sensitivity was greatly enhanced, and the peak current was obviously improved when capsaicinoids were functionalized to atp-AuNPs@Fe_3_O_4_ nanocomposites via the diazotization–coupling reaction. 

The electrochemical behaviors of capsaicinoids on the SPCE with different modifications were further characterized via DPV, as shown in Figure 3B. No apparent peaks appeared in 3.0 ng/mL capsaicin on the bare SPCE (curve a). A weak peak was discovered in 3.0 ng/mL capsaicin on the AuNPs@Fe_3_O_4_ nanocomposite modified SPCE (curve b), whereas a large oxidation peak emerged when 3.0 ng/mL capsaicin was functionalized to the atp-AuNPs@Fe_3_O_4_–nanocomposite–modified SPCE via the diazotization–coupling reaction (curve c). When the blank solution was detected on the AuNPs@Fe_3_O_4_–nanocomposite with the diazotization–coupling-reaction-modified SPCE, no obvious oxidation peaks (curve d) were observed. The results were consistent with the above detection via CV.

**Figure 4:**
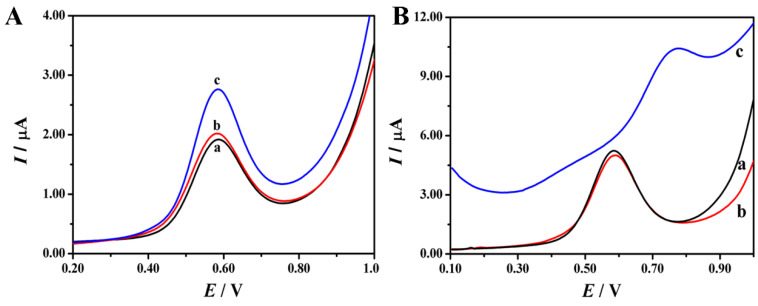
(**A**) DPV curves in the supporting electrolyte based on AuNPs/Fe_3_O_4_ nanocomposites and azo reaction by the SPCE for (a) 0.50 ng/mL capsaicin; (b) 0.50 ng/mL dihydrocapsaicin; and (c) 0.50 ng/mL capsaicin as well as 0.50 ng/mL μmol/L dihydrocapsaicin. (**B**) DPV curves for 3.00 ng/mL capsaicin based on AuNPs/Fe_3_O_4_ nanocomposites and azo reaction before and after the sample pretreatment. (a) Capsaicin standard; (b) ICO sample after LLE; and (c) ICO sample before LLE.

To explore the electrochemical behaviors of capsaicinoids on the modified SPCE in this work, capsaicin and dihydrocapsaicin were detected via DPV, as shown in Figure 4A. We detected 0.50 ng/mL capsaicin (curve a), 0.50 ng/mL dihydrocapsaicin (curve b), and a mixture of 0.50 ng/mL capsaicin and dihydrocapsaicin (curve c), respectively. The results showed that capsaicin and dihydrocapsaicin presented the same oxidation potential and similar oxidation currents. Then, the mixture of capsaicin and dihydrocapsaicin was detected, and the oxidation peak current was approximately the sum of the peak currents of the two compounds separately. Thus, the results showed that capsaicinoids had the same electrochemical behaviors. Therefore, capsaicin was used as a representative to establish and evaluate the electrochemical method for capsaicinoids in this work. 

### 3.3. Matrix Effects

In this study, LLE was used to eliminate the matrix effect interference of the ICO samples. To investigate the interference of the matrix effect, the blank oil sample and ultrapure water were both spiked with 3.00 ng/mL capsaicin. They were then analyzed via the developed electrochemical method before and after LLE, respectively. The results are shown in Figure 4B. The oxidation current of capsaicin (curve b) in the blank oil sample is slightly lower than that in the standard solution of capsaicin (curve a), and the oxidation potential is almost the same. At the same experimental conditions, the untreated oil sample was measured. It was found that the oxidation current was significantly decreased with the positive shift of the oxidation peak potential (curve c). In addition, the baseline elevated significantly. Therefore, LLE was applied to eliminate the matrix effects in ICO samples to guarantee sensitivity in the subsequent work.

### 3.4. Optimizations of the Diazotization–Coupling Reaction Conditions

The pH values of the diazotization reaction and coupling reaction were taken into account in the work, as shown in Figure S1. It was observed that the oxidation peak current of capsaicin increased gradually with the increase in the pH value from 1.0 to 3.0. The oxidation peak current of capsaicin decreased when the pH value of the diazotization reaction was higher than 3.0. This indicated that the optimal pH value of the diazotization of 4-atp was 3.0, as shown in Figure S1A.

With capsaicinoids as the alkaloids containing phenolic hydroxyl groups, the coupling reaction needs to be performed at weakly basic conditions. Therefore, we examined the pH value of the coupling reaction ranging from 8.0 to 10.5, as shown in Figure S1B. The results showed that the oxidation peak current of capsaicin increased to its maximum at pH 9.0. Therefore, pH 9.0 was used for the coupling reaction of 4-atp with capsaicinoids.

The reaction time for the coupling of 4-atp with capsaicinoids was also optimized in this study. The oxidation peak current of capsaicin increased gradually when the coupling reaction time extended from 20 to 30 min, as shown in Figure S1C, whereas the oxidation peak current of capsaicin decreased when the reaction time was more than 30 min. As the reaction time exceeded the optimal time for the coupling, the resulting compound may have decomposed, and the sensitivity of the measurement decreased. Therefore, the coupling reaction time was chosen to be 30 min.

The volume of AuNPs@Fe_3_O_4_ nanocomposites was important for the reaction. Volumes from 5.0 to 20.0 μL of the nanocomposites were examined. The oxidation current of capsaicin increased gradually with the increase in the volume of AuNPs@Fe_3_O_4_ nanocomposites from 5.0 to 10.0 μL, while the oxidation peak current of capsaicin achieved a constant value when the volume of nanocomposites was more than 10.0 μL, as shown in Figure S1D. Therefore, the volume of AuNPs@Fe_3_O_4_ nanocomposites was chosen to be 15.0 μL, considering the widest possible linear range of detection and reagent cost savings. 

### 3.5. Optimizations of the pH Value and Concentration of Support Electrolyte 

The pH value and concentration of the supporting electrolyte had a significant effect on the electrochemical behavior of the capsaicin. It showed that the oxidation peak current of capsaicinoids reached a maximum at pH 9.0, as shown in Figure S1E. The oxidation peak current of capsaicinoids increased gradually when the concentration of the K_2_SO_4_ solution increased from 0.10 to 0.20 mol/L, whereas the oxidation peak current decreased gradually when the concentration of K_2_SO_4_ solution ranged from 0.20 to 0.30 mol/L, as shown in Figure S1F. Thus, the pH value of the supporting electrolyte was selected to be 9.0, and the concentration of the K_2_SO_4_ solution was chosen to be 0.20 mol/L.

### 3.6. Optimizations of the Sample Pretreatment Conditions

As capsaicinoids are easily soluble in strong alkaline solutions, a 0.5 mol/L NaOH solution was used to pretreat ICO samples [12]. The effect of the volume of the NaOH solution on the extraction efficiency of capsaicinoids in ICO samples was examined. The results indicated that the oxidation peak current of capsaicinoids reached its maximum when the volume of NaOH was 1.2 mL, indicating that the extraction efficiency was the highest, as shown in Figure S2A.

In addition, the influences of the speed and time of the oscillator were investigated, as shown in Figure S2. At a speed of 250 rpm, the peak current response of capsaicinoids achieved the highest figure, as shown in Figure S2B. The effect of the oxidation peak current of capsaicinoids between 10 and 30 min into the shaker time was examined, as shown in Figure S2C. The results showed that the extraction efficiency of capsaicinoids increased with the extension of the extraction time, and reached its maximum at an extraction time of 20 min. Thus, a speed of 250 rpm and time of 20 min for the oscillator were adopted in subsequent analyses.

### 3.7. Performance of the Sensor

#### 3.7.1. Calibration Curve and Limit Detection

**Figure 5:**
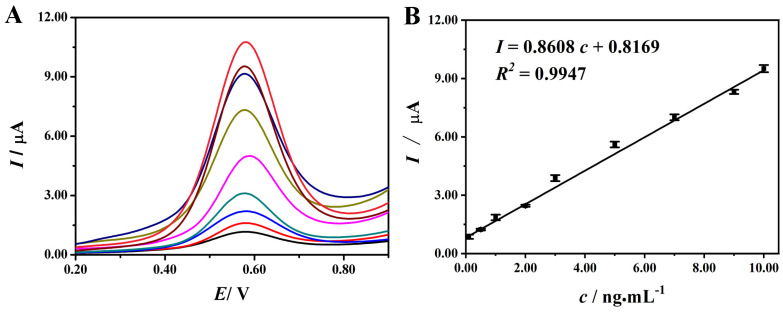
(**A**) The DPV curves for different concentrations of capsaicin (0.10, 0.50, 1.00, 2.00, 3.00, 5.00, 7.00, 9.00, and 10.00 ng/mL) in the blank oil sample detected via the SPCE. (**B**) A calibration curve depicting the relationship between current and standard capsaicin concentrations. All error bars represent standard deviations in the measurements (*n* = 5).

Different concentrations of capsaicin standards were added into the blank oil sample to prepare a series of standard solutions (the concentrations of capsaicin were 0.10, 0.50, 1.00, 2.00, 3.00, 5.00, 7.00, 9.00, and 10.00 ng/mL). At optimal conditions, the performance of the electrochemical sensor was evaluated via detection of capsaicinoids in oil samples, as shown in Figure 5. The oxidation peak current and concentration of capsaicin showed a good linear relationship in the range of 0.10 to 10.00 ng/mL. The regression equation was *I* = 0.5737 *c* + 0.5375, *R*^2^ = 0.9978. The limit of detection (LOD) was 0.02 ng/mL (S/N = 3). The limit of quantification (LOQ) was defined as the quantification at the minimum level via the stepwise dilution of low concentrations of capsaicinoids in oil samples, and was found to be 0.05 ng/mL.

#### 3.7.2. Precision

To evaluate the precision of the method, three concentrations of capsaicin standard solutions (0.30, 3.00 and 8.00 ng/mL) were added into the blank oil sample. The content of capsaicin was measured five times in a day for five continuous days. The relative standard deviations in intra-day and inter-day measurements for different concentrations of capsaicin were calculated, as shown in Table S1. The results showed that the deviations ranged from 4.9% to 5.7% for intra-day, and from 6.4% to 7.7% for inter-day, indicating an acceptable repeatability of the established method.

#### 3.7.3. Accuracy

Low, medium, and high concentrations of capsaicin standard solutions were added into the spiked oil sample and measured to assess the precision of this method. Each concentration was measured in parallel five times. The results showed that the recoveries of capsaicin at different concentrations in oil samples ranged from 92.8% to 103.9%, and the coefficient of the variations were below 7.7%, as listed in Table S2. The satisfactory recoveries indicated a good accuracy for this method. 

#### 3.7.4. Interference Test

It is reported that animal fats generally contain large amounts of cholesterol, while edible vegetable oils contain little cholesterol [35]. Therefore, cholesterol was selected as an interferent. The concentrations of capsaicinoids before and after adding interferences were defined as X_C_ and X_T_, respectively. The interference value was expressed via X_T_-X_C_. When the value was less than 1.96*S*, it indicated insignificant interference and was expressed by *N*. Meanwhile, significant interference was expressed via *I* when the value was more than 1.96*S*. 

The results showed that there was significant interference when the concentration of cholesterol was over 5.00 μg/mL, as listed in Table S3. In addition, some common ions were also investigated. The results showed that no obvious interferents were observed in 0.10 mol/L Mg^2+^, Cu^2+^, K^+^, Na^+^, Ca^2+^, Cl^−^, and SO_4_^2−^, as well as 0.01 mol/L Fe^2+^, solutions. In summary, good anti-interference capability was presented for the detection of capsaicinoids in ICO via this method. 

### 3.8. Analytical Application

In this study, three positive ICO samples and three negative control samples from the Chongqing Municipal Material Evidence Identification Center were blindly tested via both the established electrochemical method and the LC-MS/MS method [36]. The results are shown in Table 1. At present, ICO can be identified when the concentration of capsaicinoids is more than 1.00 ng/mL in oil samples [12,13]. The results obtained via the developed method were consistent with the results obtained via the LC-MS/MS method, indicating that the established method showed good accuracy and reliability for the determination of capsaicinoids in ICO.

## 4. Conclusions

A highly sensitive method for the detection of capsaicinoids was established, based on the amplification strategy of AuNPs@Fe_3_O_4_ nanocomposites and the diazotization–coupling reaction. The method combined the magnetic separation property of magnetic nanomaterials with the excellent biocompatibility of gold nanoparticles. In addition, capsaicinoids were coated and enriched on AuNPs@Fe_3_O_4_ nanocomposites via the diazotization–coupling reaction on a self-assembled 4-atp monolayer. The liquid–liquid extraction technique was used to pretreat ICO samples. Capsaicinoids were collected on the surface of the electrode to achieve the effective identification of ICO. The presented method is highly sensitive with a low cost. It provides a novel and sensitive method for the detection of capsaicinoids in the food hygiene field, which could be used for the identification of ICO. It is of great significance for ensuring food safety and protecting public health.

## Figures and Tables

**Table 1:** Detection of capsaicinoids in illegal cooking oils based on AuNPs/Fe_3_O_4_ nanocomposites and the diazotization–coupling reaction via the SPCE (*n* = 5).

Number of Illegal Cooking Oil	Concentration (Mean ± SD, ng/mL)	Result
1	-	Negative
2	-	Negative
3	-	Negative
4	4.05 ± 0.23	Positive
5	3.69 ± 0.05	Positive
6	7.32 ± 0.13	Positive

## Data Availability

Not applicable.

## Supplementary Materials

The following supporting information can be downloaded at: https://www.mdpi.com/article/10.3390/bios13090863/s1, Figure S1: Effects of different experimental conditions on the oxidation currents of capsaicin (n = 5). (A) pH value of the diazotization reaction. (B) pH value of the coupling reaction. (C) The time of the coupling reaction. (D) The volume of AuNPs/Fe_3_O_4_ nanocomposites. (E) The concentrations of K_2_SO_4_ solution. (F) pH value of the supporting electrolyte.; Figure S2: Effects of different experimental conditions for sample pretreatment of ICO on the oxidation currents of capsaicin (n = 5). (A) The volume of NaOH solution. (B) The speed of the oscillator. (C) The time of the oscillator.; Table S1: The precisions of capsaicinoids in ICO detected by the electrochemical method (n = 5).; Table S2: The recoveries of capsaicinoids in ICO detected by the electrochemical method (n = 5).; Table S3: The effect of interferences on the determination of capsaicinoids in ICO (n = 5).
