# Supplementary material for: A Highly Sensitive Electrochemical Sensor for Capsaicinoids and Its Application in the Identification of Illegal Cooking Oil
Source: Biosensors (Basel). 2023 Sep 1;13(9):863. doi: 10.3390/bios13090863 (PMC10526801; doi:10.3390/bios13090863)
Supplement: Supplementary file 1 [file biosensors-13-00863-s001.zip › biosensors-2530734-SI.pdf]

A Highly Sensitive Electrochemical Sensor for Capsaicinoids and Its Application in the Identification of Illegal Cooking Oil

Wenjing Lyu [1,†], Min Ding [1,†], Ying Zhou [1], Mengdan Jiang [1], Yanru Li [1], Yanxiang Ding [2], Zhong Zhang [3], Xue Wei [1] and Xiaoqing Zhang [1,*]

[1] Key Laboratory of Clinical Laboratory Diagnostics, Ministry of Education of China, School of Laboratory Medicine, Chongqing Medical University, Chongqing 400016, China; 305452@hospital.cqmu.edu.cn (W.L.); dingmin@cqmu.edu.cn (M.D.); 17774964041@163.com (Y.Z.); 18208371513@163.com (M.J.); 18468154207@163.com (Y.L.); 2022110551@stu.cqmu.edu.cn (X.W.)

[2] The First Clinical College, Chongqing Medical University, Chongqing 400016, China; tanktank0905@163.com

[3] Material Evidence Identification Center, Chongqing Public Security Bureau, Chongqing 400016, China; zhangzhong5123@126.com

* Correspondence: xzhang5@cqmu.edu.cn; Tel.: +86-23-68485239; Fax: +86-23-68485992

† These authors contributed equally to this work.

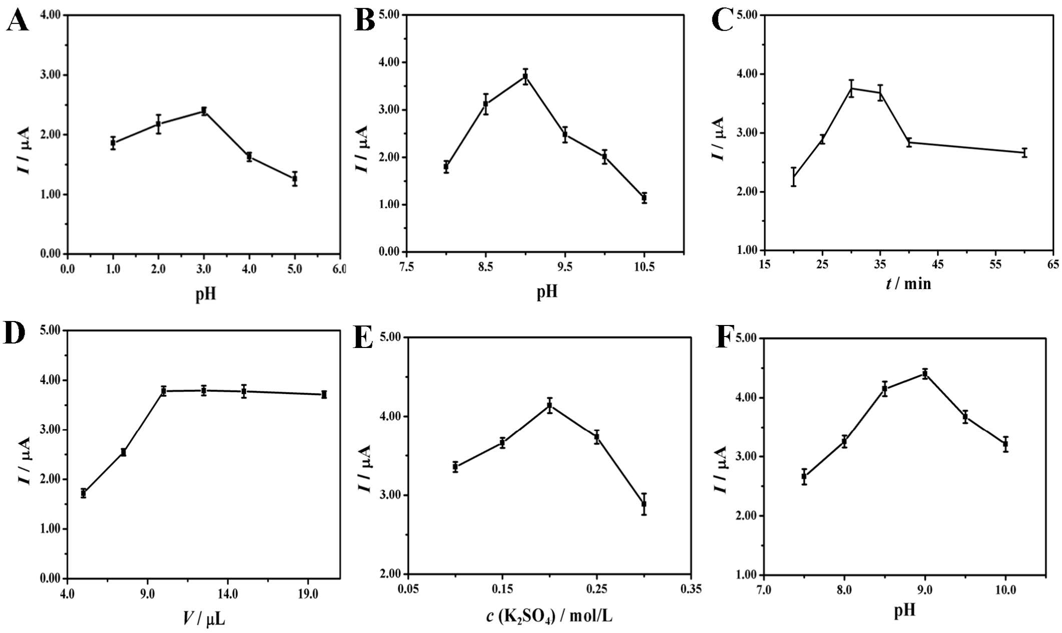

Figure S1. Effects of different experimental conditions on the oxidation currents of capsaicin (n = 5). (A) pH value of the diazotization reaction. (B) pH value of the coupling reaction. (C) The time of the coupling reaction. (D) The volume of AuNPs/Fe_3O_4 nanocomposites. (E) The concentrations of K_2SO_4 solution. (F) pH value of the supporting electrolyte.

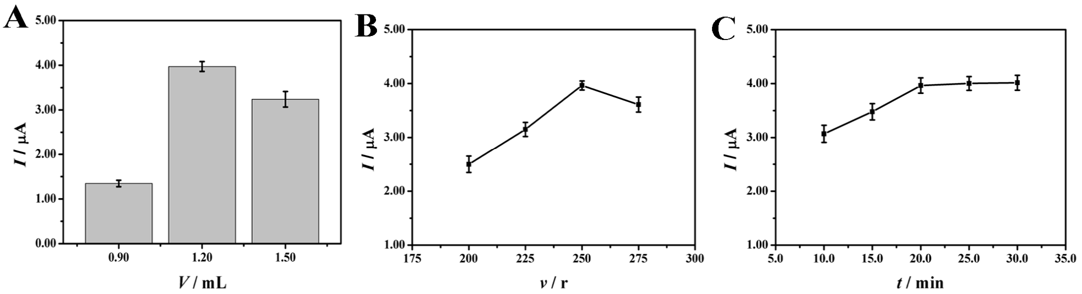

Figure S2. Effects of different experimental conditions for sample pretreatment of ICO on the oxidation currents of capsaicin (n = 5). (A) The volume of NaOH solution. (B) The speed of the oscillator. (C) The time of the oscillator.

Table S1. The precisions of capsaicinoids in ICO detected by the electrochemical method (n = 5).

Added concentration (ng/mL)	Intra-day		Inter-day	
	Measured concentration (Mean ± SD, ng/mL)	Precision (RSD, %)	Measured concentration (Mean ± SD, ng/mL)	Precision (RSD, %)
0.30	0.34 ± 0.02	5.7	0.34 ± 0.03	7.7
3.00	3.12 ± 0.15	4.9	3.12 ± 0.20	6.4
8.00	8.13 ± 0.42	5.1	8.00 ± 0.58	7.3

Table S2. The recoveries of capsaicinoids in ICO detected by the electrochemical method (n = 5).

Added concentration (ng/mL)	Sample concentration (ng/mL)	Measured concentration (Mean ± SD, ng/mL)	Precision (RSD, %)	Recovery (%)
0.30	1.09	1.37 ± 0.09	6.7	103.9
3.00	1.09	3.98 ± 0.30	7.7	96.4
8.00	1.09	9.46 ± 0.69	7.3	92.8

Table S3. The effect of interferences on the determination of capsaicinoids in ICO (n = 5)

Added cholesterol（μg/mL）	Capsaicin（ng/mL）		
	Measured	$X_T - X_C$	1.96S
0	0.62 ± 0.05		0.09
0.05	0.50 ± 0.03	-0.02	*N*
0.50	0.48 ± 0.04	-0.04	*N*
5.00	0.45 ± 0.04	-0.70	*N*
6.00	0.41 ± 0.04	-0.11	*I*
